# An organisation working mainly reactively instead of proactively: a qualitative study of how frail users of home care services and their next of kin experience crises

**DOI:** 10.1186/s12913-024-11544-5

**Published:** 2024-09-19

**Authors:** Janne Myhre, Sverre Bergh, Øyvind Kirkevold, Bjørn Lichtwarck

**Affiliations:** 1 The Research Centre for Age-related Functional Decline and Disease, Innlandet Hospital Trust, Ottestad, Norway; 2 Department of Health and Nursing Science, Faculty of Social and Health Sciences, Inland Norway University of Applied Sciences, INN University, Elverum, Norway; 3 Norwegian Centre for Violence and Traumatic stress Studies, (NKVTS), Oslo, Norway; 4 Norwegian National Advisory Unit on Ageing and Health, Vestfold Hospital Trust, Vestfold, Norway; 5 Department of Health, Care and Nursing, Faculty of medicine NTNU, Norwegian University of Science and Technology, Gjøvik, Norway

**Keywords:** Home care services, Crises, Users of home care services, Next of kin, Frailty, Community-dwelling people, Behavioural and psychological symptoms of dementia (BPSD), Complexity theory

## Abstract

**Background:**

Frail people receiving home care services face an increased risk of developing crisis, which can result in adverse events, coercive measures, and acute institutionalisation. The prevalence of frailty is expected to increase due to the ageing population in most countries. However, our knowledge of the process leading to crises among frail community-dwelling patients remains limited. The aim of our study was to explore how users of home care services and their next of kin experienced crises and how these crises were approached by home care services.

**Methods:**

A qualitative explorative design with 21 interviews was conducted. We explored crises within the last year that had led to an acute institutionalisation (hospital or nursing home) or to an unstable situation with high risk of institutionalisation. Systematic text condensation (STC) was used to analyse the data.

**Results:**

Our findings are summarised into one overarching theme; an organisation working mainly reactively instead of proactively, which is supported by four subthemes: (1) insufficient communication—a determinant of crises, (2) the lack of a holistic approach, (3) a sense of being a burden, and (4) the complexity of crises. The reactive approach is demonstrated in the participant’s experience of insufficient communication and the lack of a holistic approach from the service, but also in the user’s sense of being a burden, which seems to be reinforced by the experienced busyness from the staff in the home care services. This reactive approach to crises seems to have contributed to difficulties in detecting the various stressors involved in the complex process leading to crisis.

**Conclusions:**

Our findings suggest that home care services tend to be characterised by a reactive approach to rising instability and the development of crises for users. This can be interpreted as an emergent property of the organisation and the adaptation towards exceeding demands due to insufficient capacity in health care services. We recommend the use of multicomponent care programmes comprising interdisciplinary case conferences in home care services to implement a cultural change that can shift the service from a reactive, fragmented, and task-oriented approach to a more proactive approach.

**Supplementary Information:**

The online version contains supplementary material available at 10.1186/s12913-024-11544-5.

## Background

The risk of developing crises is particularly high in frail community-dwelling people who due to their functional decline are dependent on care from the home care services. These crises can be defined as ‘*a process where the stressors cause an imbalance requiring an immediate decision which leads to a desired outcome and therefore crisis resolution*’ [1]. Such crises can have a burdensome impact on both users of home care services, their next of kin, and care staff. The stressors that cause this imbalance, thereby triggering and maintaining crises, are heterogeneous and vary among users of home care services. Such stressors may include neuropsychiatric symptoms (NPS), rejection of care, anxiety or depression and social isolation [2]. For the users, crises often result in adverse events, coercive measures, and acute institutionalisation [3]. For the users’ next of kin, crises can lead to depression, burn-out, isolation, physical and verbal abuse, and omission of care [2]. In community-dwelling users of home care services, 1–5% are classified as high-risk patients for the development of crisis, and of these 15–35% have a particularly high risk of acute hospitalisation or acute admittance to long-term care [4].

Many of the users of home care services have multimorbidity, and they represent a heterogeneous group due to great variation in diseases, functional level and age [5, 6]. Because of an aging population in many countries, there will be a rising number and proportion of people with multimorbidity. Multimorbidity will probably double in the next 20 years, and cognitive decline, dementia and depression will affect one-third of these patients [6]. The risk of frailty increases with increasing age, and for adults 65 years or more the prevalence of frailty is estimated to 11%, increasing to 50% for adults older than 80 years [7]. A recent report issued by the Norwegian Health Directory noted that 140,648 people aged 75 years and older can be defined as frail, accounting for 31% of the total population within that age group in Norway [8]. Physiological vulnerability comprising a reduced capacity to adapt to internal and external stressors, is usually described as the core feature of the concept frailty [9]. However, lately researchers have emphasized a biopsychosocial definition of frailty, which also includes what is labelled psychological frailty and social frailty [10]. In this way, frailty is understood as a multifaceted concept where biological, psychological, and social stressors interact to cause this state of reduced capacity to adapt and manage stressors.

The literature on crises in home-dwelling people has mainly explored this phenomenon in relation to people with dementia [1, 3]. The perceptions of crises experienced by people with dementia, their next of kin and the health care staff have been found to be influenced by the various type of stressors behind the crises and where the users of home care services are living when the crises occur [1]. This underlines the complexity and diversity of the processes that leads towards crises. This complexity of the process leading towards a crisis may be one of the reasons why it remains a major challenge for the health care system to be able to provide high-quality and safe health care to frail community-dwelling patients [11]. Frail individuals face high risks of poor outcomes after hospital discharge, with increased 2-year mortality rates, compared to non-frail patients [12]. Preventing crises from leading to institutionalisation is therefore important, and interventions aimed at reducing preventable hospitalisation are needed. To develop such interventions, research is needed to explore the processes leading to crises with outcomes such as hospitalisation or other types of institutionalisations [13]. The Norwegian Health Directorate has emphasized the importance of early detecting and responding to changes in the patient’s health condition as a part of a national patient safety programme [14]. Early detection and response by health care professionals are important patient safety strategies, and have the potential to prevent crises or to alleviate the consequences of such crises when they first have occurred.

### Norwegian home care services

Home care services in Norway are part of the public health system and are provided to users free of charge [15, 16]. The main tasks for the home care services comprise professional nursing care, providing diverse medical treatment and also assistance with the users’ activities of daily life. Since these tasks are extensive, varied and complex, and since the home care services in their work are dependent on a close collaboration with general practitioners (GPs), hospitals, other primary health care workers, and the social assistance sector etc., the home care services can be considered as complex organisations [5]. In most Norwegian municipalities, home care services are organised based on a purchaser-provider model that splits such services into care units and administrative units [17]. The administrative unit makes multiple single decisions regarding the type of health care and assistance that are necessary for the applicant [15, 16]. These single decisions define the care tasks the user will receive from the home care services and outline how many minutes the home care services will use for each task. These services are organised based on worklists that describe in detail the users that home care services staff must visit, the tasks that they must perform, and the number of minutes that are allocated to each task [17, 18].

### Aim of the study

The study aimed to explore (i) how users of home care services and their next of kin experienced crises and (ii) how users of home care services and their next of kin perceived the response of home care services in managing those crises.

## Methods

We used a qualitative explorative design featuring individual in-depth interviews of users of home care services and their next of kin. Because the questions and topics on which this study focuses are related to vulnerable personal situations resulting from critical situations, individual interviews were perceived as the most appropriate method for this research. Qualitative methods provide “contextual knowledge” concerning people’s experiences of the situations they face and the ways in which they interpret, understand, and link meaning to events [19]. By collecting information from the perspectives of both users of home care services and their next of kin, the intention of the present study was to develop a deeper understanding of crises from the perspective of the users. This study follows the Consolidated Criteria for Reporting Qualitative Research (COREQ) [20].

### Settings and participants

This study is part of the larger Preventing and approaching crises for frail community-dwelling patients through innovative care study (PRACTIC), which involves 30 municipalities, and their home care services in all health care regions in Norway. The 30 municipalities in the main PRACTIC project were recruited from April until December 2022 (Dalbak E.T, Væringstad A, Lichtwarck B, Myhre J, Holle D, Bergh S, et.al., Preventing and Approching Crisis for Frail community- dweling Patients Through Innovative Care (PRACTIC):study protocol for a process evaluation of a complex intervention in home care service/submitted) [21]. The sample used in the present study was recruited from nine of the 30 municipalities, including both urban and rural areas. Home care service users and their next of kin were recruited using a stepwise approach from January until July 2023, as we employed a process of theoretical sampling until data saturation was achieved [22]. This stepwise approach involves a continuous parallel process of analysing interviews and recruitment of informants until no new information emerges. The selection of these nine municipalities was based on factors related to conveniences, such as travel distance and economy; in addition, we did not want to interfere with the randomised controlled trial (RCT) conducted as part of the PRACTIC study (i.e., we did not include users of home care services who were already included in the RCT) (Dalbak ET, Væringstad A, Lichtwarck B, Myhre J, Holle D, Bergh S, et.al., Preventing and Approching Crisis for Frail commuity-dweling patient Through Innovative Care (PRACTIC): study protocol for a process evaluation of a complex intervention in home care service /submitted)  [21].

Within these nine municipalities, purposive sampling was used to ensure that the participants recruited met the following inclusion criteria: (1) in need of home care services and (2) a score ≥ 5 on the Clinical Frailty Scale (CFS) (indicating mild, moderate, or severe frailty) [23]. In addition, (3) the users were required to have experienced a crisis during the past year, which was defined as (a) having been admitted to acute care in the hospital, (b) having been admitted to acute care in a nursing home, or (c) being perceived by home care service staff as facing an unstable situation featuring a high risk of acute institutionalisation or having exhibited rejection of care. The exclusion criteria for users were as follows: (1) planned hospitalisation or nursing home placement, (2) the presence of a clear single medical cause of institutionalisation (e.g., stroke or hip fracture), or (3) short life expectancy (i.e., < 4 weeks). The inclusion criteria for next of kin were as follows: (1) identity as next of kin of a user of home care services who met the inclusion criteria mentioned above and (2) regular contact with the user in question (i.e., at least once per week).

A total of twenty-one interviews were conducted two months to one year after a crisis had occurred among seventeen users of home care services. The users of home care services and their next of kin decided whether they wanted to be interviewed together or separately. All participants were interviewed only once. The interviews were conducted in the following manner:

- Five interviews were conducted with only users as participants; these users did not want to involve their next of kin (5 users interviewed).
- Seven interviews were conducted jointly with the user and the next of kin, who functioned as a dyad (7 users and 7 next of kin interviewed).
- Six interviews were conducted with three users and three of their next of kin separately (3 users and 3 next of kin interviewed).
- One interview was conducted solely with one user’s next of kin. This user was interviewed with another next of kin as a dyad (1 next of kin interviewed).
- Two interviews were conducted with only the next of kin as participants; the users were not interviewed due to their deteriorating state of health. Information concerning the users and the crises they faced was obtained (2 next of kin interviewed).

These twenty-one interviews included interviews with thirteen next of kin and fifteen users. The two interviews conducted with the next of kin without the presence of the users involved information concerning the users and the crises they faced; hence, the characteristics of 17 users and 13 next of kin are described in Table 1.

**Table 1:** Characteristics of the users of home care services and their next of kin

Interviews (*n* = 21) with 15 users of home care services and 13 of their next of kin were conducted. Two interviews with the next of kin were conducted without the corresponding users but involved information about those users hence, 17 users are described.		
	**Users of home care services (*n*** **= 17)**	**Next of kin (***n* **= 13)**
**Age**		
< 50		1 (7.7)
50–60		7 (53.8)
61–70		3 (23.1)
71–80	3 (17.6)	2 (15.4)
81–90	11 (64.8)	
91–100	3 (17.6)	
**Gender**		
Female	15 (88.2)	8 (61.5)
Male	2 (11.8)	5 (38.5)
**Relatives’ relation to user**		
Spouse		2 (15.4)
Son		4 (30.7)
Daughter		6 (46.2)
Other		1 (7.7)
**Clinical Frailty Scale (CFS)** ^**a**^		
5	6 (35.3)	
6	5 (29.4)	
7	6 (35.3)	
**Clinical Dementia Rating Scale (CDR)** ^**b**^		
0	5 (29.4)	
0.5	8 (47.1)	
1	3 (17.6)	
2	1 (5.9)	
**Marital status**		
Living alone: divorced or never married	2 (11.8)	
Living alone: widow/widower	12 (70.6)	
Married	3 (17.6)	
**Place of residence at the time of the interview**		
Nursing home	5 (29.4)	
Home (house)	6 (35.3)	
Home (flat)	6 (35.3)	
**Type of municipality (** ***n*** ** = 9)**		
Urban area	4 (45)	
Rural area	5 (55)	

Notes: Values are shown as numbers, and percentages are shown in parenthesis. ^a^The CFS ranges from 1–9, with higher scores indicating more severe frailty (5: mild frailty, 6: moderate frailty, and 7: severe frailty). ^b^The CDR ranges from 0–3 (0: no dementia, 0.5: questionable dementia, 1: mild dementia, 2: moderate dementia, 3: severe dementia). The participants were drawn from nine municipalities in Norway

### Data collection

All interviews with the users were performed at the participant’s current location: 12 interviews were conducted at their home, and three interviews were conducted in a nursing home. Six interviews were performed with users’ next of kin via Microsoft Teams. Each interview lasted between 30 min and 90 min depending on the health status of the user and his or her capacity to participate. The mean length of the interviews was 60 min. The first author conducted all the interviews. Before the interviews were conducted, the home care service informed the researcher about the time frame of the crisis, the main causes, whether the crisis involved acute institutionalisation in a nursing home or hospital or whether it was instead an unstable situation featuring a high risk of institutionalisation. The home care services also used the Clinical Frailty Scale (CFS) [23] and the Clinical Dementia Rating Scale (CDR) [24] to assess the users who participated in this research. The CFS ranges from 1 to 9, with higher scores indicating more severe frailty (5: mild frailty, 6: moderate frailty, and 7: severe frailty). The CDR focuses on the stages of possible dementia, ranging from 0 to 3 (0: no dementia, 0.5: questionable dementia, 1: mild dementia, 2: moderate dementia, 3: severe dementia). No other information regarding the crisis was conveyed to the researcher before the interviews were conducted.

The experience of the crisis was the main topic of the interviews with all participants. The interview guide was developed for this study and has previously not been published elsewhere (see attachment 1.) The interview guide included questions about participant’s description of the situation, the events that occurred, the participant’s feelings and thoughts, the participant’s beliefs regarding the causes of the situation, ways in which the crisis could ultimately have been prevented, and the manner in which the crisis was approached. These main questions were followed up by open-ended and exploratory questions. The participants were encouraged to speak freely, and when other key themes emerged spontaneously during the interviews, time was allotted to allow participants to elaborate on these themes. The interviews were recorded and transcribed verbatim, and they were cross-checked by listening to the recorded interviews.

### Data analysis

The analysis was performed simultaneously by the first and last authors using an inductive, continuous, and iterative process based on systematic text condensation (STC), a method of thematic cross-case analysis that consists of four steps [25]. After each interview, the first and last authors read the interviews transcripts separately to obtain an overall impression, and they met to discuss any topics that required further exploration during subsequent interviews. When no new topics arose, theoretical saturation in the categories was achieved, and no new participants were recruited for further interviews [22].

The four steps of the analytical process were as follows. First, the transcripts were read several times to obtain an overall impression and develop preliminary themes, subthemes, and emerging categories that could reflect important meanings across the participants’ experiences. These preliminary themes were also assessed in relation to preliminary topics and situations that had previously been identified during the interview process. Second, meaning units, i.e., short extracts of the text that were viewed as supporting the preliminary themes, were then condensed into summarising codes. Meaning units and codes representing different aspects of the main theme and subthemes were identified. The similarities and differences among the codes were discussed, and the final codes were chosen. Third, the preliminary subthemes were reframed based on the similarities and common semantic aspects exhibited by each code group. Fourth, the condensation pertaining to each of the code groups was summarised to develop a general description and concepts reflecting the experience of crisis with an overarching theme and four final subthemes. Throughout the iterative analysis process, the researchers continually iterated among the abstracted results, i.e., the codes, the subthemes, and the main text.

### Ethical considerations

The Regional Committee for Medical and Health Research Ethics in Eastern Norway (REC South-East) approved the study (Project No. 221019). Each participant received oral and written information from the home care service staff regarding the study. Approximately a week later, the users that wanted to participate handed a signed consent form to the home care service. All identifiable characteristics were excluded from the presentation of the data to ensure the anonymity of all participants. Patients who had the capacity to provide consent were asked to provide written consent. Specifically trained staff in the hospitals, nursing homes, or home care services (depending on the patient’s location) assessed the patients’ capacity in this respect. With regard to patients who were believed to lack the capacity to consent, their next of kin were informed about the research and asked to provide consent on the patient’s behalf. Informed consents were obtained from all participants prior to the interview.

## Results

The characteristics of the users of home care services and their next of kin, such as their age, gender, degree of frailty, and possible dementia, as well as the relationship between the user and their next of kin, are presented in Table 1. Among the 17 users of home care services, fifteen were female and fourteen were aged 80 years or older, two had a spouse as their next of kin, and ten had a son or daughter as their next of kin and twelve users were living alone at the time of the crisis. Table 2 presents the characteristics of the 17 cases of crisis experienced by the 17 users of home care services based on the interviews with the 28 participants in our study and on information collected from the staff in the home care services. Among the 17 cases of crisis, two cases resulted in an acute admission to a nursing home, eleven cases resulted in acute admission to a hospital, and four cases involved an unstable situation featuring a high risk of institutionalisation. For the unstable situation, the users and their next of kin perceived a different timeframe and starting point of the crises than the researcher had been informed by the home care services. The participants found it difficult to describe the development of the unstable situations, and determining their starting points was especially difficult.

**Table 2:** Characteristics of the 17 cases of crisis

	**Number of cases (n=17)**
**Triggers of crisis: **The main categories for various types of crises based on the inclusion criteria.	
**Acute admission to nursing home**	**2 (11.8)**
*Repeated falls*	*1 (50.0)*
*Reduced ADL function*	*1 (50.0)*
*Behavioural and psychological symptoms of dementia*	*1 (50.0)*
**Acute admission to hospital**	**11 (64.7)**
*Infection*	*6 (54.5)*
*Reduced general condition*	*6 (54.5)*
*Delirium*	*1 (9.1)*
*Severe falls*	*5 (45.4)*
*Breathing difficulties*	*3 (27.2)*
*Anxiety*	*3 (27.2)*
*Pain*	*2 (18.2)*
*Poor nutrition and dehydration*	*1 (9.1)*
*Reaction to medication*	*1 (9.1)*
*Heart failure*	*1 (9.1)*
**Unstable situation**	**4 (23.5)**
*Anxiety*	*2 (50.0)*
*Loneliness*	*3 (75.0)*
*Poor nutrition*	*1 (25.0)*
*Infection*	*1 (25.0)*
*Reduced general conditions*	*2 (50.0)*
*Rejection of care*	*2 (50.0)*
*Falls*	*3 (75.0)*

Notes: The characteristics of the 17 cases of crisis as experienced by the 17 users of home care services are based on the interviews with the 28 participants in our study and on information collected from the staff in the home care services. Values are shown as numbers, and percentages are shown in parentheses. Due to the complexity of crises, one type of crisis often involves several triggers; therefore, the sum of the percentages of triggers can be more than one hundred.

The analysis revealed one overarching theme, i.e., an organisation working mainly reactively instead of proactively, and four subthemes: (1) insufficient communication—a determinant of crises, (2) the lack of a holistic approach, (3) a sense of being a burden, and (4) the complexity of crises.

### An organisation working mainly reactively instead of proactively

The main theme, “an organisation working mainly reactively instead of proactively,” referred to the overall experiences of the users of home care services and their next of kin concerning the reasons underlying crises. The approach that we labelled “reactive” reflects an abstraction of participants’ descriptions of responses to changes in patient health status, which were frequently late, and the difficulties in uncovering the complex process underlying the crisis. The reactive approach taken by the home care service was based mainly on the demands expressed by the patients, and the services failed to develop their own agenda for assessing the patients’ status. This approach often led to actions being taken only once the crisis had already occurred, such as by contacting the hospital for acute admission. On the other hand, a proactive approach based on regular observations and assessments of patients could reduce the likelihood of crisis. The four subthemes expand on this main theme.

#### Insufficient communication—A determinant of crises

All the next of kin explicitly expressed that insufficient communication was a factor that contributed to the development of crises. The insufficient communication they experienced was connected to the fact that changes in health conditions were not communicated by the home care services to either the user or the next of kin; such communication also did not occur among the staff working for the home care services or between the home care services and the patient’s general practitioner (GP). The home care services involve visits to the same user by several health care staff. This situation was conveyed as one of the main causes for the fact that changes in health conditions were not always uncovered and hence not communicated. In addition, several next of kin also questioned the ways in which these changes, if they were detected, were communicated, followed up, and documented in the electronic health records (EHR).

*A stable staff group visiting her will presumably be able to pick up signals about a change in her health status earlier because they know her. But if they are just visiting her occasionally*,* they may not know what the starting point was. However*,* you can question the communication within the home care services and what they write in the EHR. Is it such as this: ‘Now there is a change we need to focus on. She is more tired*,* eating less*,* has slept a lot.’ That is an interesting question.* (Next of kin, 2)

Insufficient communication among the home care services, the user and the next of kin was also conveyed as contributing to unclear responsibilities, especially when the health situation of the user deteriorated. Several next of kin described their uncertainty regarding whether they or the home care services were responsible for contact with the GP. This lack of clear responsibilities led the next of kin to describe themselves as coordinators who focused on communicating the user’s needs.

*“Next of kin play a coordinator role to a much greater extent than we should because the home care services do not view it as their task*,* and I just have to do it for my mother’s sake. So*,* whether it is booking an appointment or follow-up when my mother feels that she is not getting her point across or being heard*,* I take charge.”* (Next of kin, 12).

The users of home care services, on the other hand, talked about the lack of communication they experienced more implicitly. This was expressed through lack of knowledge of their health status and the fact that they did not know how changes in their health situation were followed up. The communication was often not tailored to the user’s reduced hearing and vision. This factor was also identified as leading to insufficient communication between the home care service staff and the users. In addition, the users also conveyed their concern with not knowing when the home care staff would visit them. They often had trouble contacting the home care service when they wanted to convey a message or request help:

*“The time of appointments are challenging. They say that they will arrive then and then*,* but they don’t. So*,* something what could have been better is the opportunity to reach them*,* because sometimes they just don’t answer.”* (User, 7).

#### The lack of a holistic approach

Both the users and their next of kin highlighted the lack of a response to needs other than physical health care needs, such as social and psychological needs. This factor was identified as contributing to unstable situations or acute institutionalisation. Several participants highlighted the use of single decisions as a barrier to a comprehensive approach. The lack of a response to the needs of the user of home care services was perceived as a main factor in the development of the crisis:

*“I knew that she was not feeling well psychologically. Therefore*,* we asked the home care services if they could stay and talk to her for like 10 minutes as a daily routine. However*,* this was not possible because it is not something you can apply for; it is not something they offer as a single decision*,* even though that was what she needed.”* (Next of kin, 11).

The users of home care services talked about how the different difficulties they encountered in their everyday lives gave rise to concerns, anxiety, and low quality of life, which persisted until a crisis occurred. These concerns included, for example, their inability to clean the house properly, change a light bulb, or empty the trash can themselves or their inability to open the window themselves in the summer heat in addition to their fears of current loneliness, future loneliness, or their failure to receive help when they needed it:

*“The loneliness we experience within society now makes us insecure. Because if you are alone and the home care services are the only ones you see*,* and they are always in a rush*,* then you are just sitting alone all day long. So*,* what if the heat is like it is now*,* and you are unable to open the window and let the heat out*,* or you are unable to clean the house*,* or you are out of food*,* if the house falls into disrepair around you*,* and you are staying there because you have to?”* (User, 15).

Users’ next of kin also expressed their concern for the needs and changes in health care status that were not addressed by the home care services. The next of kin described their concerns regarding fire, low nutritional status, repeated falls, and the lack of practical help; they also indicated that their loved one was alone too frequently, thus leading to loneliness and anxiety. Since not all needs were met by the home care services, all participants relied on neighbours, friend, or next of kin to live safely at home. Some participants also mentioned that they paid someone to be sure that they would receive help when they needed it or to ensure that all their needs were met. Many next of kin also mentioned that they increased their visits when a change in the health care status of their loved ones occurred:

*“As she got worse*,* we had to be there more often to be sure that she got all the help she needed.”* (Next of kin, 1).

#### A sense of being a burden

All users of home care services described their feeling of being a burden because they were dependent on help from others, such as the home care service, their family or their friends, to manage their everyday lives. Being dependent on help from others was expressed as leading to feelings of shame. On the other hand, these feelings led to a desire to remain independent and manage their health care problems and everyday lives themselves without asking for help. This desire for independence was described as a reason why users sometimes rejected care from home care services, which may have contributed to the development of crises:

*“The point is that she wants to be independent and manage everything herself*,* and she says so too: ‘I can do it myself.’ Then*,* she often says that she manages more than she actually does. So*,* when the home care service offers her help*,* she refuses.”* (Next of kin, 7).

This sense of being a burden when users needed help from others was partly identified a cultural phenomenon that users learned during their childhood and through their life experiences:

*“I had a very good mother*,* but she was forty-four years old when she had me on top of the other nine children. When she got sick*,* we looked after her for many years*,* but she was always saying things about not bothering her or not bothering him. ‘No*,* I don’t want to bother you.’ So*,* maybe it is something you learn*,* that we should not bother each other.”* (User, 14).

This culturally learned sense of being a burden and the desire to remain independent were also connected to social views of ageing and the need for help as individuals age.

*“I have a feeling that some people look at older people who are in need of help as a nuisance.”* (User, 3).

This sense of being a burden was also expressed as being reinforced by perceived signals of busyness from the home care services. All the users and their next of kin emphasised the fact that the home care services featured too few staff members. They had heard about the lack of staff in health care services from the media and from staff themselves who discussed their busy schedules. However, the participants also described busyness in the context of their experiences with home care services, such as situations in which they were not seen as persons and situations in which follow-up regarding changes in the user’s health status was lacking. This situation was also perceived by the users based on the nonverbal signals conveyed by staff when they, e.g., continually looked at their watches or failed to remove their jackets during their visits on when the phones that the staff brought with them were ringing and beeping continually during their visits.

*But I see it*,* and I know that they* [the staff working in the home care services] *are in a hurry. So*,* then I think that I can do it myself instead of asking them.* (User, 1)

All participants emphasized this sense of being a burden as a reason why the users of home care services hesitated to ask for more help and hence often received less help than they needed.

#### The complexity of crises

**Fig. 1:**
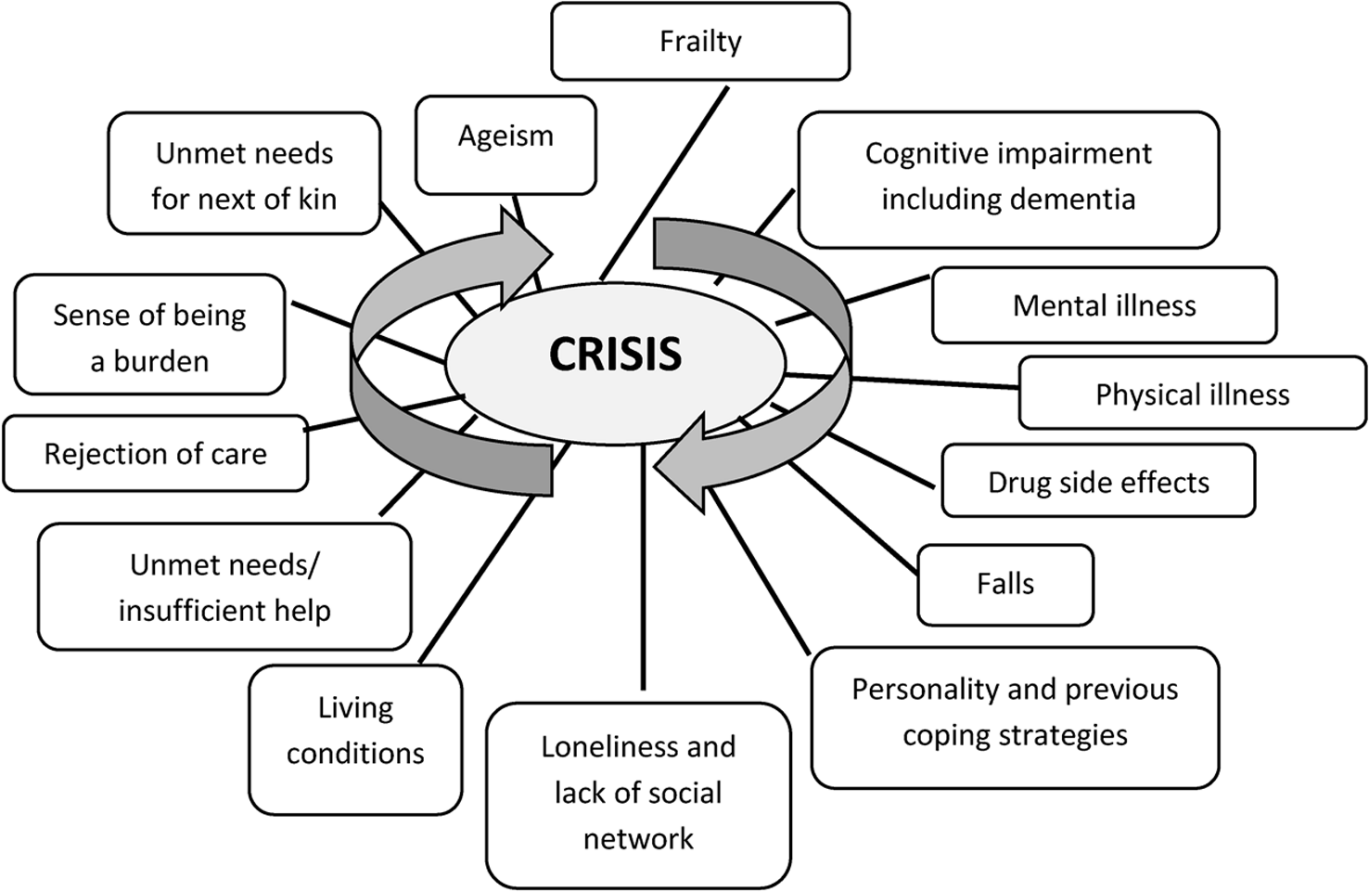
The complexity of crisis. Note: The figure summarizes the participants’ experiences of different determinants of crisis, abstracted as concepts by the authors. These determinants represent possible risk factors, triggers, and maintenance factors of a crisis, which often overlap and influence each other, displaying the complexity of crises

All participants noted that several simultaneously triggers and causes were often responsible for one acute admission to a nursing home or a hospital or to the user’s unstable situation. This highlights the multifaceted character of crises. Figure 1 shows the different determinants mentioned by the participants, abstracted as concepts by the authors. These concepts will be further developed in the discussion section. As shown in this figure, all participants identified biological, psychological, and social factors as determinants of crises. The complexity within the crisis was revealed through their description of how these determinants overlap, influence, and reinforce each other. For example, the side effects of medication were pointed out as leading to repeated falls, which in turn led to pain and anxiety, thereby resulting in poor nutrition and poor health as well as reinforcing users’ unstable gait and exacerbating the risk of falls; in addition, infections often emerged, leading to acute admission.

*“After this severe fall*,* she went to the hospital*,* where they took away these pills that she was reacting to. In addition*,* she was very dehydrated*,* thin*,* and anxious.”* (Next of kin, 10).

“*She had been tired for a while*,* and she didn’t eat much. I think that she was in great pain*,* and she was very worried and scared. She had several falls*,* so now she had an armchair that she lived in.”* (Next of kin, 13).

However, although both users of home care services and their next of kin described repeated falls or low nutritional status as factors that contributed to crises, all participants were unsure of the degree to which the users were examined and assessed with regard to these conditions. Some participants also related the user’s falls to their advanced age and expressed that they did not expect them to be examined.

*“But then*,* she turns 88 years in July*,* so in a way*,* it’s also a bit natural that she has poor balance and then falls*.” (Next of kin, 2).

## Discussion

**Fig. 2:**
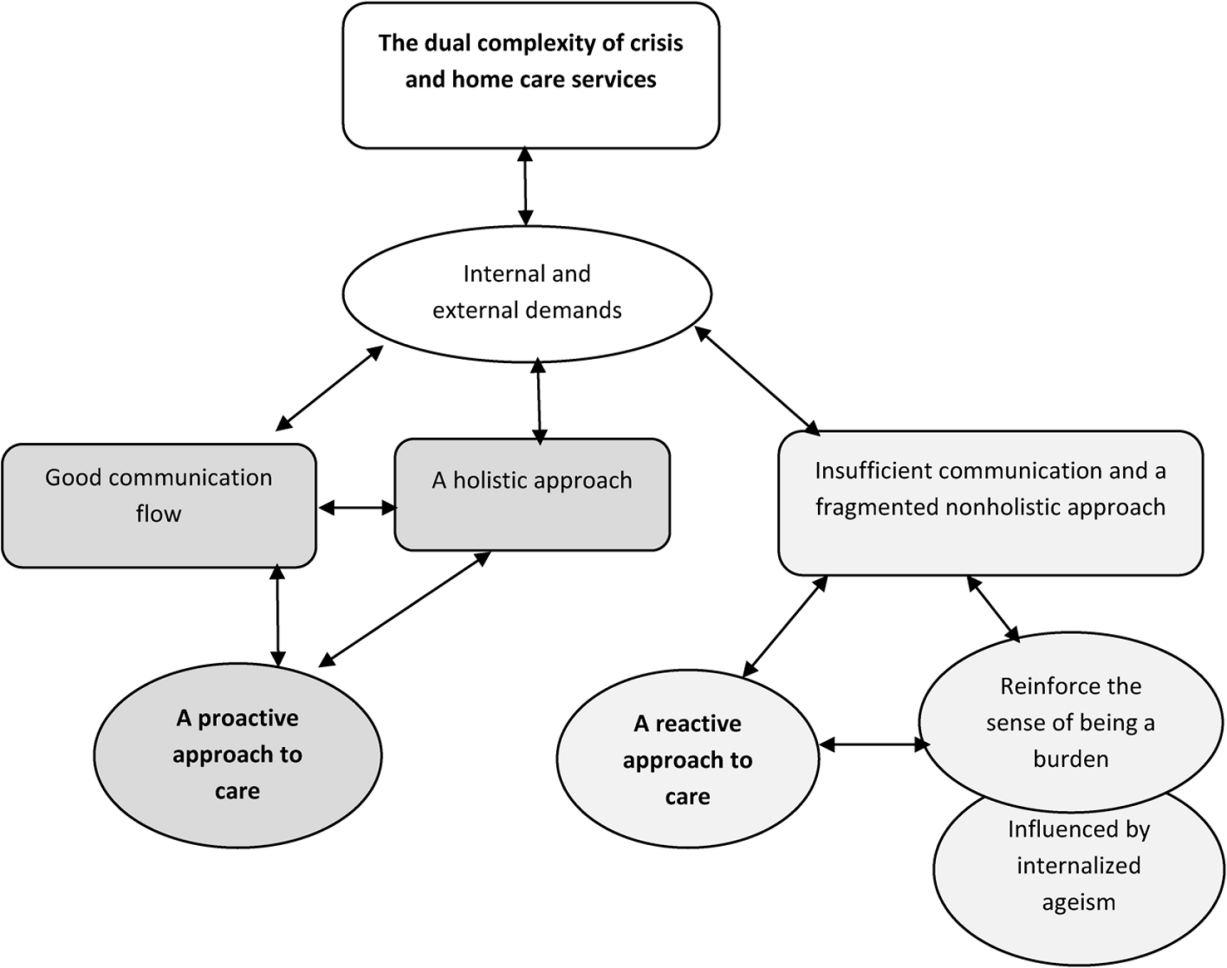
An organisation working mainly reactively instead of proactively. Note: The figure illustrates the adaption i.e., the self-organization towards a proactive or a reactive approach as a response to the dual complexity in the home care services and the users

Our findings can be summarised in terms of one overarching theme: an organisation working mainly reactively instead of proactively. This reactive approach is demonstrated not only by the participant’s experience of insufficient communication and the lack of a holistic approach on the part of the service but also by the user’s sense of being a burden, which seems to be reinforced by their experiences of busyness on the part of staff working for the home care services. This reactive approach to a crisis contributed to difficulties with regard to identifying the different stressors involved in the complex process leading to the crisis. Here, we discuss these findings in light of theories drawn from complexity science [26, 27] and the concept of “wicked problems” [28]. We will also discuss how the participants’ sense of being a burden, to a large extent, can be viewed as the consequence of the lack of a holistic approach, the lack of sufficient communication and an internalised discourse of ageism (see Fig. 2). The use of theory in qualitative research can serve as an expression of abstraction, thus enhancing our understanding of how related phenomena are connected beyond the level permitted by a mere description of the phenomena in question [29, 30].

###  Insufficient communication and the lack of a holistic approach to the complexity of crises

The complexity of the crises experienced by frail community-dwelling people and the need for a holistic approach towards such crises were evident in our findings. As shown in Fig. 1, biological, psychological, and social factors were identified by the participants as determinants of such crises. The participants experienced a lack of response from the home care services when it came to needs beyond physical health care. A longitudinal study conducted by Teo et al. to examine the dimension of frailty among community-dwelling people exhibited empirical clinical relevance when physical, mental, and social dimensions were included in the description of frailty [10]. Frailty among the users of home care services and the development of crises can be viewed as the results of complex interactions between biological, psychological, and social determinants [27, 31]. According to complexity theory, this implies that these interactions are often non-linear with a certain unpredictability in the development of crises [32]. These determinants can interchangeably be risk factors, triggers, and maintenance factors of a crisis. For example, frailty increases the risk of physical illness, which in turn is a risk factor for mental illness, reinforced by loneliness and poor living conditions; and vice versa, mental illness increases the risk for physical illness, and both factors can lead to crises.

In our context, the complex needs of frail users of home care services cause these individuals to be prone to instability [10, 27], and when such instability emerges, it ultimately culminates in a crisis. The unpredictable interactions between these factors were displayed by the participants’ difficulties to establish a starting point for the crisis and describing how the crisis developed. In this way, a crisis can be viewed as an example of a “wicked problem,” as described by Rittel and Webber [28]. These problems are interwoven with each other; they exhibit complex and overlapping causes and are difficult to delineate or define. Wicked problems are characterised by the fact that they have no natural starting or stopping point; i.e., they are not solved once and for all, are instable and require constant effort to manage [28]. Therefore, such a crisis cannot easily be defined with the aim of providing a “true” definition because each process leads to a specific crisis, and each user is different and unique [10].

In addition to the complexity of the phenomenon crisis, a dual complexity emerges, as home care services can be described as complex systems [33–35]. The complexity of the user’s needs and the complexity comprising different stakeholders around each user, such as professionals, next of kin, managers, policymakers and technologies, all of which interact with each other, account for this dual complexity. These adaptive systems rely on trade-offs and adjustments to succeed in everyday clinical work [26]. The participants highlighted insufficient communication and the lack of a holistic approach on the part of the home care services as factors that could explain why changes in the user’s health condition were not always uncovered. The fact that several health care staff members visited the same user was perceived as a primary barrier to uncover and communicate changes. If changes were not uncovered, they could not be communicated. Complexity theory describes a complex system as a system that adapts to internal and external demands, and that this behaviour is dynamic and nonlinear, exhibiting a varying degree of instability [32, 35]. Studies on patient safety have documented how adaptive actions can solve problems and prevent harm [36, 37]; however, they have also indicated that adaptability can have negative consequences if the system does not balance demands with capacity in the context of a constant effort to provide sound and safe patient care [38–40]. This adaptation is known as self-organisation in a complex system [32]. Members of an organisation, such as home care services, develop social knowledge related to routines, norms and rules related to daily life, which involves adaptation to demands and capacity, such as the acceptance of different staff members visiting the same user, the ways in which they communicate and the lack of a holistic approach [32, 38, 40]. Furthermore, the participants noted that the organisation of health care within the home care service was based on single decisions regarding physical needs, which was perceived as a barrier to meeting the actual needs of the users. In the light of complexity theory, single decisions can be viewed as a linear and simple approach to complex problems due to their task-oriented and fragmented character [41]. As previously described, frailty and the process leading to crises constitute complex or wicked problems, which consist of many determinants that are biological, psychological, or social and that interact with each other; furthermore, in this context, new challenges emerge continually over the course of the day, week, or month [26, 28]. This implies that regular professional judgements and comprehensive assessments are necessary to uncover changes in each user’s health situations [35]. Several users experienced a fall during the process of a crisis, but none had experienced an examination aimed at detecting the possible reasons underlying their falls.

Self-organisation, i.e., adaptation to internal and external demands, also occurs among users and their next of kin because these actors are in a constant dynamic relationship with each other and the home care service. When they encountered insufficient communication from the service, users’ next of kin adapted and emerged as a coordinator for communication. The lack of a holistic approach on the part of the service caused all participants to rely on neighbours, friends or next of kin to be able to live safely at home. Many next of kin increased their visits when a change in the health care status of their loved one occurred. According to complexity theory, these visits from the next of kin can be regarded as attractors to the complexity of the frailty users [26]. Attractors are factors in complex systems that contribute to increased stability and order [32]. In our study, these attractors could have caused the users to be able to remain safely at home for longer periods.

### A sense of being a burden

The participants also conveyed how the users’ sense of being a burden contributed to the development of the crisis. The burden of care for informal and formal caregivers has been acknowledged as a concept for decades [42, 43]. Although care is provided in the context of a dynamic relationship, less attention has been given to care recipients’ sense of being a burden to others. A systematic review of the end-of-life experiences of dependent patients revealed an overall increase in their feelings of being a burden to others [44]. These feelings involved frustration and guilt due to the hardships they imposed on their caregivers. In a study of suicide among older people, living as a burden was identified as a factor that can explain why older people commit suicide [45]. Living with such a sense of being a burden was found to be connected to functional decline and illness among older persons [44, 45].

The participants in our study identified the sense of being a burden they experienced and their desire to remain independent as social phenomena they had learned through their life experiences. The tendency to strive for independency even in need of help can be interpreted as a learned coping strategy and as a part of the user’s personality [46]. In addition, society’s negative views of ageing and being a person who is dependent on care, which were reinforced through the signals of busyness from the home care service, enhanced this sense of being a burden. With regard to ageism [47], such a sense of being a burden can be viewed in connection to the ways in which society perceives and treats older people. Ageism refers to how we think (stereotypes), how we feel (prejudices), and how we act (discrimination) towards people as a result of their age [47]. In contemporary society, values such as independence, youthfulness, productivity, effectiveness, and coping are located at the top of the value hierarchy [45, 47]. This society identifies positive values with independence and people’s ability to cope with challenges on their own, thus implying that dependency and vulnerability can be viewed as negative factors. The most appreciated values within a society affect (often in an implicit manner) how we think, feel, and act towards older people, i.e., the discourse on old age that characterizes the society in question [47, 48]. This discourse can be insidiously internalised by older people and affect how older people think and feel regarding themselves [45, 47, 48].

The signals of busyness from staff seem to have reinforced users’ sense of being a burden and were a factor that participants highlighted to explain why the users did not receive the help they needed. This finding implies that the actions of staff members express their adaptation in the home care services, which among other factors, may be influenced by their competence, attitudes as well as low staffing, and the culture of which they are a part. This adaption is also influenced by societal attitudes towards ageing and dependency [33, 35, 46]. Furthermore, it implies that the actions taken by users, such as avoiding asking for help, are ways of adapting to the users’ experiences of busyness on the part of staff working for the home care services as well as society’s attitudes towards dependency and vulnerability.

### An organisation working mainly reactively instead of proactively - Implications for practice

All the participants in our study expressed a need for what we have termed a proactive approach from these services. A proactive approach stands in contrast to an approach based on a system of single decisions that is entailed by the purchaser-provider model in many municipalities. Fragmented single decisions can be perceived as both a consequence of a reactive approach and an important factor maintaining such a reactive task-oriented approach.

For home care services to display a proactive approach to crises, the organisation must have the ability to discover changes in the user’s health during their everyday work [38, 40]. The implication for practice for the home care services is to develop routines with a focus on daily professional judgements based on regular systematic comprehensive assessments, joint reflection, and effective communication routines.

This self-organisation observed in complex organisations such as home care services, which was here shown to result in a mainly reactive approach towards users, cannot be controlled; rather, it can only be influenced [32, 38]. According to Stacy [49], three conditions influence such self-organisation in complex organisations: (1) increasing information flow, (2) facilitating more contact points between the participants in the organisation, and (3) promoting the development of greater variety in terms of cognitive schemas (interpretation and understanding). One suggestion for implementing a shift from a mainly reactive to a more proactive approach is the use of systematic multicomponent care programmes based on regular assessments and interdisciplinary reflection involving case conferences [35, 50]. These case conferences have the potential to influence self-organisation in terms of all three of these aspects [35, 46]. The use of multicomponent care programmes in home care services may therefore contribute to a cultural shift from a reactive and fragmented, task-oriented service to a more proactive, holistic approach. As part of our research project, i.e., the PRACTIC study, we are now testing the effectiveness of a multicomponent model called TIME through an RCT conducted in the context of home care services (Dalbak ET, Væringstad A, Lichtwarck B, Myhre J, Holle D, Bergh S, et.al., Preventing and Approching Crisis for Frail community -dweling Patient Through Innovative Care (PRACTIC): study protocol for a process evaluation of a complex intervention in home care service / submitted) [21]. TIME is a manual based programme that includes a rigorous assessment of the crisis, one or more interdisciplinary case conferences and the testing and evaluation of customised treatment measures [50, 51].

### Strengths and limitations of the study

This study involved 21 interviews with users of home care services and their next of kin, from nine different municipalities in Norway. This approach increases the transferability of our findings. By collecting information from the perspectives of both users of home care services and their next of kin, the aim of the present study was to develop a deeper understanding of the crisis from the users’ perspectives. This approach strengthens the credibility and trustworthiness of the study. The research team has used a biopsychosocial approach to frailty to get a broader and more comprehensive understanding of this concept. This approach could have contributed to a richer description of different determinant of crisis. However, it may also have increased the transferability of our findings to the heterogeneous population of frail users of home care services. All interviews were conducted two months to one year after a crisis had occurred. A limitation of this research is that it can be difficult to remember events that occurred a year ago. However, all the crises reported in this research, with two exceptions, represented situations that had occurred during the past six months. The experienced crises seemed to be situations associated with strong emotional impacts on the participants, which may have strengthened their memories of the events. Since both users and their next of kin were included in most of the interviews, they also helped each other recall the crises.

The first author of this study, JM, is a nurse as well as a researcher and has previously worked in home care services; however, none of those home care services were involved in this study. Because of this background knowledge, it was possible to pose in-depth questions to explore a broad range of issues. However, background knowledge could also affect the type of follow-up questions that were asked during the interviews. To counterbalance this potential bias, two researchers, JM and BL (a physician with particular competence in old age and nursing home medicine), analysed the data continuously beginning with the first interview using the STC analytical method, which made it possible to develop and deepen the questions asked during the interviews further. The STC analytical method is clearly described and can easily be replicated by others, thus contributing to the transparency of our study.

## Conclusions

Our findings indicate that homecare services are characterised by a mainly reactive approach towards instability and crises on the part of users. This can be interpreted as an emergent property of health care service self-organisation, i.e., the adaptation of these services to internal and external demands. The reactive approach to a crisis contributes to difficulties in identifying the different stressors involved in the complex process leading to the crisis. A proactive approach, on the other hand, involves professional judgement on a daily basis, regular systematic comprehensive assessments and effective communication concerning changes in a user’s health condition. We recommend the use of multicomponent care programmes including interdisciplinary case conferences in home care services to contribute to a cultural shift from a reactive, fragmented, task-oriented approach to service to a more proactive approach.

## Supplementary Information

Supplementary Material 1.

## Data Availability

The datasets generated and analysed as part of the current study are not publicly available due to the fact that the format of the data does not allow for complete anonymisation. However, the data are available from the corresponding author upon reasonable request.

## Abbreviations

CDR: Clinical Dementia Rating Scale
CFS: Clinical Frailty Scale
EPR: Electronic health records
GP: General practitioner
PRACTIC: Preventing and Approaching Crises for Frail Community-dwelling Patients Through Innovative Care
RCT: Randomised controlled trial
SCT: Systematic Text Condensation
TIME: Targeted Interdisciplinary Model for Evaluation and Treatment of Neuropsychiatric Symptoms

## References

[1] Vroomen JM, Bosmans JE, van Hout HP, de Rooij SE. Reviewing the definition of crisis in dementia care. BMC Geriatr. 2013;13:10. 23374634 10.1186/1471-2318-13-10 PMC3579755

[2] Stolp T, Brown M, Toevs S, Berlin M. Impact of dementia behavioral crises events on first responder and family systems in Idaho: an exploratory study. Boise, US: Boise State University; 2016.

[3] Ledgerd R, Hoe J, Hoare Z, Devine M, Toot S, Challis D, et al. Identifying the causes, prevention and management of crises in dementia. An online survey of stakeholders. Int J Geriatr Psychiatry. 2016;31(6):638–47. 26489696 10.1002/gps.4371

[4] International Global Forum for Health Care Innovators. Mind the gap; managing the rising-risk patient population 2016. https://www.advisory.com/topics/high-risk-patient-management/2017/12/mind-the-gap-managing-the-rising-risk-patient-population

[5] Genet N, Boerma W, Kroneman M, Hutchinson A, Saltman R. Home care across Europe: current structure and future challenges: World health organization: Regional Office for Europe; 2012. https://apps.who.int/iris/bitstream/handle/10665/327948/9789289002882-eng.pdf

[6] Kingston A, Robinson L, Booth H, Knapp M, Jagger C. Projections of multi-morbidity in the older population in England to 2035: estimates from the population ageing and care simulation (PACSim) model. Age Ageing. 2018;47(3):374–80. 29370339 10.1093/ageing/afx201 PMC5920286

[7] Collard RM, Boter H, Schoevers RA, Voshaar RCO. Prevalence of frailty in community-dwelling older persons: a systematic review. J Am Geriatr Soc. 2012;60(8):1487–92. 22881367 10.1111/j.1532-5415.2012.04054.x

[8] Helsedirektoratet. Bruk av tjenester i kommunenen og somatisk sykehus blandt skrøpelige eldre [The use of services in municipalities and hospitals among frail older people]. 2023 https://www.helsedirektoratet.no/rapporter/bruk-av-tjenester-i-kommunene-og-somatiske-sykehus-blant-skropelige-eldre

[9] Travers J, Romero-Ortuno R, Bailey J, Cooney MT. Delaying and reversing frailty: a systematic review of primary care interventions. Br J Gen Practice: J Royal Coll Gen Practitioners. 2019;69(678):e61–9. 10.3399/bjgp18X700241 PMC6301364 30510094

[10] Teo N, Yeo PS, Gao Q, Nyunt MSZ, Foo JJ, Wee SL, et al. A bio-psycho-social approach for frailty amongst Singaporean Chinese community-dwelling older adults - evidence from the Singapore Longitudinal Aging Study. BMC Geriatr. 2019;19(1):350. 31830924 10.1186/s12877-019-1367-9 PMC6909571

[11] Di Pollina L, Guessous I, Petoud V, Combescure C, Buchs B, Schaller P, et al. Integrated care at home reduces unnecessary hospitalizations of community-dwelling frail older adults: a prospective controlled trial. BMC Geriatr. 2017;17(1):53. 28196486 10.1186/s12877-017-0449-9 PMC5310012

[12] Keeble E, Roberts HC, Williams CD, van Oppen J, Conroy SP. Outcomes of hospital admissions among frail older people: a 2-year cohort study. Br J Gen Practice: J Royal Coll Gen Practitioners. 2019;69(685):e555–60. 10.3399/bjgp19X704621 PMC6650131 31308000

[13] Shepherd H, Livingston G, Chan J, Sommerlad A. Hospitalisation rates and predictors in people with dementia: a systematic review and meta-analysis. BMC Med. 2019;17(1):130. 31303173 10.1186/s12916-019-1369-7 PMC6628507

[14] Helsedirektoratet. Tidlig oppdagelse og rask respons ved forverret somatisk tilstand: Nasjonale faglige råd; 2020. https://www.helsedirektoratet.no/faglige-rad/tidlig-oppdagelse-og-rask-respons-ved-forverret-somatisk-tilstand

[15] Helse-og omsorgsdepartementet. Lov om kommunale helse-og omsorgstjenester m.m. [the municipalities health and care service act] 2011. https://lovdata.no/dokument/NL/lov/2011-06-24-30

[16] Helse-og omsorgsdepartementet. Forskrift om egenandel for kommunale helse-og omsorgstjenester [regulation on deductibles for municipal health and care services] 2011.

[17] Holm SG, Mathisen TA, Sæterstrand TM, Brinchmann BS. Allocation of home care services by municipalities in Norway: a document analysis. BMC Health Serv Res. 2017;17(1):673. 28938892 10.1186/s12913-017-2623-3 PMC5610450

[18] Strandås M, Wackerhausen S, Bondas T. Gaming the system to care for patients: a focused ethnography in Norwegian public home care. BMC Health Serv Res. 2019;19(1):121. 30764824 10.1186/s12913-019-3950-3 PMC6376668

[19] Patton MQ. Designing qualitative studies. In: Patton M, editor. Qualitative research and evaluation methods. Los Angeles: Sage; 2015. pp. 230–46.

[20] Tong A, Sainsbury P, Craig J. Consolidated criteria for reporting qualitative research (COREQ): a 32-item checklist for interviews and focus groups. Int J Qual Health Care. 2007;19(6):349–57. 17872937 10.1093/intqhc/mzm042

[21] Anette Væringstad ET, GjelsethDalbak D, Holle J, Myhre Øyvind, Kirkevold S, Bergh, et al. Preventing and approaching crises for frail community-dwelling patients through innovative care (PRACTIC): protocol for an effectiveness cluster randomised controlled trial. Trials. 2024;25(1):304. 38711048 10.1186/s13063-024-08117-6 PMC11075302

[22] Charmaz K. Constructing grounded theory: a practical guide through qualitative analysis. Thousand Oaks, California: SAGE; 2006.

[23] Rockwood K, Song X, MacKnight C, Bergman H, Hogan DB, McDowell I, et al. A global clinical measure of fitness and frailty in elderly people. CMAJ: Can Med Association J = J de l’Association medicale canadienne. 2005;173(5):489–95. 10.1503/cmaj.050051 PMC1188185 16129869

[24] Berg L. Clinical dementia rating (CDR). Psychopharmacol Bull. 1988;24(4):637–9. 3249765

[25] Malterud K. Qualitative research: standards, challenges, and guidelines. Lancet (London England). 2001;358(9280):483–8. 11513933 10.1016/S0140-6736(01)05627-6

[26] Cilliers P. Complexity and Postmodernism: understanding complex systems. London: Routledge; 1998.

[27] Cilliers P. Complexity and Postmodernism: understanding complex systems. New York, NY: Taylor & Francis; 2002.

[28] Rittel HWJ, Webber MM. Dilemmas in a general theory of planning. Policy Sci. 1973;4(2):155–69.

[29] Malterud K. Theory and interpretation in qualitative studies from general practice: why and how? Scand J Public Health. 2016;44(2):120–9. 26647095 10.1177/1403494815621181

[30] Laksov KB, Dornan T, Teunissen PW. Making theory explicit - an analysis of how medical education research(ers) describe how they connect to theory. BMC Med Educ. 2017;17(1):18. 28103854 10.1186/s12909-016-0848-1 PMC5248446

[31] Engel GL. The need for a new medical model: a challenge for biomedicine. Volume 196. New York, NY: Science; 1977. pp. 129–36. 4286. 10.1126/science.847460 847460

[32] Cilliers P. Self-organisation in complex systems. Complexity and Postmodernism. London: Routledge; 1998.

[33] Anderson RA, Issel LM, McDaniel RRJ. Nursing homes as complex adaptive systems: relationship between management practice and resident outcomes. Nurs Res. 2003;52(1):12–21. 12552171 10.1097/00006199-200301000-00003 PMC1993902

[34] Lichtenstein BB, Uhl-Bien M, Marion R, Seers A, Orton JD, Schreiber C. Complexity leadership theory: an interactive perspective on leading in complex adaptive systems. Dep Manag: Fac Publ. 2006;8(4):2–12.

[35] Lichtwarck B, Myhre J, Goyal AR, Rokstad AMM, Selbaek G, Kirkevold Ø, et al. Experiences of nursing home staff using the targeted interdisciplinary model for evaluation and treatment of neuropsychiatric symptoms (TIME) - a qualitative study. Aging Ment Health. 2019;23(8):966–75. 29669442 10.1080/13607863.2018.1464116

[36] Berg SH, Aase K. Resilient characteristics as described in empirical studies on health care. In: Wiig S, Fahlbruch B, editors. Exploring resilience: a scientific journey from practice to theory. Cham: Springer International Publishing; 2019. pp. 79–87.

[37] Ellis LA, Churruca K, Clay-Williams R, Pomare C, Austin EE, Long JC, et al. Patterns of resilience: a scoping review and bibliometric analysis of resilient health care. Saf Sci. 2019;118:241–57.

[38] Anderson JE, Ross AJ, Macrae C, Wiig S. Defining adaptive capacity in healthcare: a new framework for researching resilient performance. Appl Ergon. 2020;87:103111. 32310111 10.1016/j.apergo.2020.103111

[39] Anderson JE, Ross AJ, Back J, Duncan M, Snell P, Walsh K, et al. Implementing resilience engineering for healthcare quality improvement using the CARE model: a feasibility study protocol. Pilot Feasibility Stud. 2016;2:61. 27965876 10.1186/s40814-016-0103-x PMC5154109

[40] Braithwaite J, Herkes J, Ludlow K, Testa L, Lamprell G. Association between organisational and workplace cultures, and patient outcomes: systematic review. BMJ open. 2017;7(11):e017708. 29122796 10.1136/bmjopen-2017-017708 PMC5695304

[41] Cilliers P. Approaching complexity. Complexity and Postmodernism. London: Routledge; 1998.

[42] Bruvik FK, Ulstein ID, Ranhoff AH, Engedal K. The effect of coping on the burden in family carers of persons with dementia. Aging Ment Health. 2013;17(8):973–8. 23614391 10.1080/13607863.2013.790928

[43] Bastawrous M. Caregiver burden–a critical discussion. Int J Nurs Stud. 2013;50(3):431–41. 23131724 10.1016/j.ijnurstu.2012.10.005

[44] McPherson CJ, Wilson KG, Murray MA. Feeling like a burden to others: a systematic review focusing on the end of life. Palliat Med. 2007;21(2):115–28. 17344260 10.1177/0269216307076345

[45] Kjølseth I, Ekeberg O, Steihaug S. Why suicide? Elderly people who committed suicide and their experience of life in the period before their death. Int Psychogeriatr. 2010;22(2):209–18. 19747423 10.1017/S1041610209990949

[46] Lichtwarck B, Myhre J, Selbaek G, Kirkevold Ø, Rokstad AMM, Benth J, et al. TIME to reduce agitation in persons with dementia in nursing homes. A process evaluation of a complex intervention. BMC Health Serv Res. 2019;19(1):349. 31151437 10.1186/s12913-019-4168-0 PMC6544967

[47] World Health Organization. Global report on ageism: global campaign to combat ageism. World Health Organization; 2021.

[48] Foucault M. Discipline and Punish: the birth of the prison. St Ives: Penguin Books; 1977.

[49] Stacey RD. The space for creativity in an organization. Complexity and creativity in organizations. San Fransisco: Berrett-Koehler; 1996. pp. 165–91.

[50] Lichtwarck B, Selbaek G, Kirkevold Ø, Rokstad AMM, Benth J, Lindstrøm JC, et al. Targeted interdisciplinary model for evaluation and treatment of neuropsychiatric symptoms: a cluster randomized controlled trial. Am J Geriatric Psychiatry: Official J Am Association Geriatric Psychiatry. 2018;26(1):25–38. 10.1016/j.jagp.2017.05.015 28669575

[51] Myhre J, Lichtwarck B. How and why does it work? A video-based qualitative analysis of case conferences to reduce BPSD through the lens of Habermas’s theory of communicative action. BMC psychiatry. 2024;24(1):520. 39039488 10.1186/s12888-024-05959-x PMC11265080

