# Supplementary material for: An organisation working mainly reactively instead of proactively: a qualitative study of how frail users of home care services and their next of kin experience crises
Source: BMC Health Serv Res. 2024 Sep 19;24:1102. doi: 10.1186/s12913-024-11544-5 (PMC11414183; doi:10.1186/s12913-024-11544-5)
Supplement: Supplementary file 1 — Supplementary Material 1. [file 12913_2024_11544_MOESM1_ESM.docx]

**What is the characteristics of crises in frail community-dwelling people receiving home care services?**

**Interview guide for users of home care services and their next of kin.**

Main Quentin 1:

How do users of home care services and their next of kin describe crises?

1. Some time ago you/ your loved one - were urgently admitted to a nursing home/ hospital or were in a situation where there was a risk for acut admission [adapt to the individuel participant and if it is a users or next of kin].

**Can you tell me about this situation, what happened?**

Eloboration of this question, if the participant does not get into it:

1. *How did you experience the situation? [what did actually happened, i.e. the course of events itself, thoughts and feelings about what happened]*
2. *What was important to you in this situation?*
3. *What do you think caused the situation?*
4. *Is there anything you wish was done differently before the situation arose?*
5. *How did you contact your GP/ the home care services to get help in this situation?*
6. *How was this contact answered?*
7. **Is there anything you think could have been done to prevent the situation that occurred?**

Eloboration of this question, if the participant does not get into it:

1. Is there anything about your/ your loved ones living situation or social situation that could have been done to prevent this situation?
2. Is there anything the health care services could have done to prevent the situation?
3. Is there anything you [the user and next of kin] could have done to prevent the situation?

Main Question 2

How do users of home care services and their next of kin describe the handling of the crisis?

1. **Can you tell me how you experienced that the situation we just have talked about was handled?**

Elaboration of this question if the participants does not get in to it:

1. How did you experience that the health care services handled the situation?
2. How did you experience that your close one handled the situation?
3. How did you experience that you handled the situation yourself?
4. **What worked well in the way this situation was handled?**
5. **Do you think that something could have been done in a different way?**
